# Must

**Published:** 1892-06-11

**Authors:** 


### Passing Topics.

### "MUST."

We sincerely hope that Mr. Walter Besant's well reached conclusion to the introduction he has written to Mr. Egmont Hake's book on "Suffering London," will be less cynically received by those to whom it is addressed than was the plea of the struggling Frenchman who, when he predicated that a man must live, received a quiet reply to the effect that the necessity was not obvious.

In regard to the hospitals, it appears no more necessary to give in order to live, than it is to live in order to give, because things are so topsy-turvy that the great majority of men and women share the advantages of our hospital system without contributing to its support, while a few individuals exhibit but a posthumous benevolence, which, lest we be misunderstood, we hasten to add, is only less valuable than it might be, because it too often begins, and necessarily ends, with the act of bequest.

It is a plain truth that were it not for the legacies they receive many of the hospitals of London must close their doors, and no one in the hospital world ought to say anything but what is grateful and respectful of testamentary donations. None the less, it is for the gifts from the living rather than the dead hand that hospitals hunger. They plead for the warm and instant sympathy which would lead every member of the community to regard a share in their work as his birthright.

Mr. Irving, while recently advocating the claims of a particular hospital, has reminded us how few of all the ills which men endure, lie in Kings' power to lessen or to cure, and without any disparagement to the truly regal benefactions of some of our richer men and women, we may appropriately appeal to the power of the shillings and the pence to make up the deficit attendant upon "Charity by Cheque."

Hospital Sunday, the great opportunity of the year for all classes of donors, actual and potential, is at hand. The small givers might find it difficult to single out particular hospitals for help, but by means of the Sunday Fund they may benefit all. Its coffers are large and deep, and however thickly they may be lined with cheques and notes and the other emblems convenient to people whose wealth is too weighty for the pocket, there is room and to spare for those metallic realities, which, whether they be of gold or silver or bronze, so they be faithful representatives of the giver's powers, possess a significance, a sanctity. and a value all their own.

It has been asserted before, but iteration is often the only argument wherewith to reach deaf ears, that relatively to the population, our hospitals' needs are little indeed. Thanks to the bounty of earlier generations and the care and ability displayed in fostering particular institutions, more than one-half the annual expenditure is provided for. Mr. Besant has set forth succinctly the several reasons why men should do their part towards supplying the remainder. They appear difficult to resist, and perhaps it is to the Hospital Sunday Fund of this and succeeding years that we must look to give us the best indication of their practical efficacy. Mr. Besant's is a master's hand. It is capable of awakening responsive notes in human hearts. Let us trust that the last chord he has struck in his hospital symphony will linger long and loud, and that many who have hitherto refrained will begin to give because they must.

Agricola.

